# Closing the Psychological Treatment Gap During the COVID-19 Pandemic With a Supportive Text Messaging Program: Protocol for Implementation and Evaluation

**DOI:** 10.2196/19292

**Published:** 2020-06-22

**Authors:** Vincent Israel Opoku Agyapong, Marianne Hrabok, Wesley Vuong, April Gusnowski, Reham Shalaby, Kelly Mrklas, Daniel Li, Liana Urichuk, Mark Snaterse, Shireen Surood, Bo Cao, Xin-Min Li, Russ Greiner, Andrew James Greenshaw

**Affiliations:** 1 Department of Psychiatry Faculty of Medicine and Dentistry University of Alberta Edmonton, AB Canada; 2 Addiction and Mental Health Alberta Health Services Edmonton, AB Canada; 3 Department of Psychiatry Cumming School of Medicine University of Calgary Calgary, AB Canada; 4 Strategic Clinical Networks System Innovation and Programs Alberta Health Services Calgary, AB Canada; 5 Department of Community Health Sciences Cumming School of Medicine University of Calgary Calgary, AB Canada; 6 Department of Computing Science Faculty of Science University of Alberta Edmonton, AB Canada; 7 APEC Digital Hub for Mental Health Edmonton, AB Canada

**Keywords:** COVID-19, Text4Hope, mobile phones, text, anxiety, depression, stress, pandemic, e-mental health

## Abstract

**Background:**

Coronavirus disease (COVID-19) has spread globally with far-reaching, significant, and unprecedented impacts on health and everyday life. Threats to mental health, psychological safety, and well-being are now emerging, increasing the impact of this virus on world health. Providing support for these challenges is difficult because of the high number of people requiring support in the context of a need to maintain physical distancing. This protocol describes the use of SMS text messaging (Text4Hope) as a convenient, cost-effective, and accessible population-level mental health intervention. This program is evidence-based, with prior research supporting good outcomes and high user satisfaction.

**Objective:**

The project goal is to implement a program of daily supportive SMS text messaging (Text4Hope) to reduce distress related to the COVID-19 crisis, initially among Canadians. The prevalence of stress, anxiety, and depressive symptoms; the demographic correlates of the same; and the outcomes of the Text4Hope intervention in mitigating distress will be evaluated.

**Methods:**

Self-administered anonymous online questionnaires will be used to assess stress (Perceived Stress Scale), anxiety (Generalized Anxiety Disorder-7 scale [GAD-7]), and depressive symptoms (Patient Health Questionnaire-9 [PHQ-9]). Data will be collected at baseline (onset of SMS text messaging), the program midpoint (6 weeks), and the program endpoint (12 weeks).

**Results:**

Data analysis will include parametric and nonparametric techniques, focusing on primary outcomes (ie, stress, anxiety, and depressive symptoms) and metrics of use, including the number of subscribers and user satisfaction. Given the large size of the data set, machine learning and data mining methods will also be used.

**Conclusions:**

This COVID-19 project will provide key information regarding prevalence rates of stress, anxiety, and depressive symptoms during the pandemic; demographic correlates of distress; and outcome data related to this scalable population-level intervention. Information from this study will be valuable for practitioners and useful for informing policy and decision making regarding psychological interventions during the pandemic.

**International Registered Report Identifier (IRRID):**

DERR1-10.2196/19292

## Introduction

### Background

Coronavirus disease (COVID-19), a severe acute respiratory syndrome caused by the SARS-CoV-2 virus (severe acute respiratory syndrome coronavirus 2; officially identified in January 2020 in Wuhan, China), is now a global pandemic with far-reaching, significant, and unprecedented impacts on human health and everyday life. The World Health Organization declared the COVID-19 outbreak a Public Health Emergency of International Concern [1] on January 30, 2020, with many countries globally struggling to adapt to its impact. The closing of schools and small and large businesses, extremely high unemployment rates, and the effects of quarantine are further stressors facing the global population due to COVID-19 [2].

Threats to mental health, psychological safety, and well-being are now emerging, increasing the impact of this virus on world health [3,4]. Over half of survey respondents in China rated the psychological impact of COVID-19 as moderate or severe, with 29% reporting significant anxiety symptoms and 17% reporting significant depressive symptoms [5]; these symptoms persisted after 4 weeks of the COVID-19 epidemic [6]. A number of factors may correlate with psychological impact, including female gender, student status, specific physical symptoms (eg, myalgia, dizziness, and coryza), and poor self-rated health status. A recent rapid review of 24 published studies on pandemics reported negative psychological effects, including posttraumatic stress symptoms, confusion, and anger [7]. Stressors included longer quarantine duration, infection fears, frustration, boredom, inadequate supplies, inadequate information, financial loss, and stigma.

In a study focused on health care workers (HCWs), over half had significant symptoms of depression, approximately 45% showed significant anxiety symptoms, and one-third experienced sleep disturbance and insomnia [8]. Correlates of symptomatology were related to exposure (eg, working in Wuhan, working on the front line) and demographic factors, including gender and occupation (eg, female, nurse). Although an important and inevitable public health measure during a highly infectious disease outbreak, quarantine is associated with a number of negative psychological and social effects (eg, posttraumatic stress, anger, fear, financial loss, and stigma) [7], and may serve as an additional risk factor. The literature describing the psychological impact of natural disasters suggests that a subset of people exposed to natural disasters struggle with clinically significant mental health conditions, including anxiety, depression, and substance use disorders [9-11]. Several risk factors were identified for the development of psychological conditions after disasters. In addition to the demographic factors described above, these include degree of exposure [12-16], gender [17-20], social stressors (eg, unemployment status [17] or low socioeconomic status [18]), as well as pre-existing mental health conditions [18,21,22].

Even at this early stage of the global pandemic, there is evidence of significant psychological effects among the general population, which may be more pronounced in certain groups (eg, female, socially stressed, frontline worker, pre-existing psychological disorder) [23,24]. Providing support for these challenges is difficult because of the high number of people requiring support in the context of a need to maintain physical distancing.

Mobile health technology offers a unique and innovative solution in this context. Specifically, this tool offers a convenient, cost-effective, and accessible means for implementing population-level interventions. Almost 90% of Canadians own a smartphone [25], and SMS text messaging is free to end users, does not require technical skill for use, and does not require expensive data plans. Text messages are also cost-effective to providers, costing cents per message to deliver.

Supportive text messages are associated with positive outcomes, including the reduction of depressive symptoms, increased abstinence duration in alcohol use disorder, and high user satisfaction, as reported in previous research. For example, in randomized controlled trials (RCT), patients with depression that received supportive text messages showed symptom reduction on a standardized self-report when compared to a similar patient group that did not receive text messages (with large effect sizes: Cohen *d*=0.85, Cohen *d*=0.67) [26,27]. In another RCT, to evaluate the effectiveness of an addiction-related supportive SMS text messaging mobile intervention in improving treatment outcomes for patients with alcohol use disorder, small to moderate effects were found for the cumulative abstinence duration. In addition, the intervention group's mean time to first day to drink was over twice the length of that of the control group (60 versus 26 days, respectively) [27]. In two user satisfaction surveys, over 80% of subscribers reported that a supportive SMS text messaging program improved their mental health [27,28]. Subscribers reported text messages made them feel more hopeful about managing issues (82%), in charge of managing depression and anxiety (77%,), and connected to a support system (75%); in addition, such messages improved their overall mental wellbeing (83%) [27].

### Objective

This protocol describes the implementation of the Text4Hope program (a low-cost, evidence-based, supportive SMS text messaging service) in Canada. The objective of the project is to implement a self-subscribing daily supportive text message program (Text4Hope) to close the psychological treatment gap and reduce anxiety and stress related to the COVID-19 crisis among Canadians. Our research questions include the following: (1) What are the prevalence rates of stress, anxiety, obsessive compulsive, and depressive symptoms in Canada related to the COVID-19 crisis? (2) What are the demographic correlates of stress, anxiety, obsessive compulsive, and depressive symptoms? (3) Will the Text4Hope program help reduce stress, anxiety, and depressive symptoms among Canadians experiencing psychological distress as a result of the COVID-19 crisis?

## Methods

### Evaluation Methodology and Measurement Plan

In the Text4Hope program, individuals self-subscribe to receive daily supportive text messages for 3 months by texting “COVID19HOPE” to 393939. The messages are aligned with a cognitive behavioral framework, with content written by mental health therapists as well as our research team members (authors MH and VIOA). The following is an example of the messages sent: “When bad things happen that we can’t control, we often focus on the things we can’t change. Focus on what you can control; what you can do to help yourself (or someone else) today” [29]. The messages are preprogrammed into an online software that delivers messages at 9 AM each morning. At the onset of the first message, respondents are welcomed to the service and are invited to complete an online baseline survey capturing demographic information; COVID-19–related self-isolation/quarantine information; and responses on the Generalized Anxiety Disorder-7 (GAD-7) scale [30], Perceived Stress Scale [31], and the Patient Health Questionnaire-9 (PHQ-9) [32]. Survey questions were programmed into SelectSurvey.net, an online survey tool operated by the Alberta Health Services Evaluation Services Team. No incentives are offered to respondents. Participation in the program is entirely voluntary, and completion of the survey was not a prerequisite requirement to receive supportive text messages. Subscribers may opt out at any time by texting “STOP” to 393939. Survey responses will be stored within our regional health system (Alberta Health Services) Select Survey account, and data will be exported, stored, and maintained by the Research and Evaluation team within our health region. The supportive SMS text messaging project subscriber recruitment plan was based on the success of a Text4Mood program in Alberta that was launched in response to the Fort McMurray wildfire disaster in 2016. Text4Hope has been the subject of a wide-exposure communications campaign (TV, radio, internet, and print media), including the local provincial mental health foundation, the single provincial government health care provider Alberta Health Services (AHS). Additionally, Text4Hope was the subject of a specific COVID-19 mental health support media release by the Provincial Chief Medical Officer [33]. Ethics approval has been granted by the University of Alberta Health Research Ethics Board (Pro00086163).

### Sample Size Considerations

Based on previous experience using the technology, (ie, >10,000 recipients within 6 months), we expect about 300,000 Canadians to subscribe to the Text4Hope program over the next 6 months. Based on a response rate of 21.7% for our prior Text4Mood survey [27], we anticipate around 20,000 responses to the Text4Hope surveys per 100,000 subscribers.

### Outcome Measures

The primary outcome is changed scores at 6 and 12 weeks from baseline on the Perceived Stress, GAD-7, and PHQ-9 scales. The secondary outcomes are the following: (1) changes in prevalence rates for perceived stress, anxiety, and depression from the early phase of the COVID-19 pandemic to a later phase, as measured with the Perceived Stress, GAD-7, and PHQ-9 scales, respectively; (2) the interaction between primary outcomes and the demographic characteristics of subscribers as well as the date of subscription to Text4Hope relative to the phase of the pandemic in Alberta; and (3) subscriber satisfaction/experience.

### Proposed Timeline and Milestones

The first stage involved the creation and review of the supportive text messages (targeting stress and anxiety-related concerns to COVID-19), and the programming of the messages into the software. This stage was completed on March 20, 2020. The second stage involved the launch of the Text4Hope program, which occurred on March 23, 2020. The remainder of the project will be focused on data analysis and reporting.

### Hypotheses

Our hypotheses, based on previous research, are as follows: (1) High rates of stress, anxiety, and depression will be reported, affecting one-third to half of the general population; the 1-week prevalence rates for these disorders will increase as the pandemic continues, compared to rates in the early phase. (2) Specific risk factors will be found for the experience of distress during the pandemic, such as female gender, risk of exposure, and social determinants of health (eg, employment, housing). (3) The intervention will result in a 25% or greater reduction in perceived stress, anxiety, and depressive symptoms (as measured by the Perceived Stress, GAD-7, and PHQ-9 scales) at 6 and 12 weeks from baseline. (4) At least 80% of subscribers will express satisfaction with the Text4Hope program and perceive the daily supportive text messages as contributing to their overall mental well-being.

## Results

Project evaluation will proceed using the Reach, Effectiveness, Adoption, Implementation, and Maintenance (RE-AIM) Framework [34] and the Alberta Quality Matrix for Health [35]. Specifically, dimensions considered will include the following: acceptability (subscriber satisfaction/experience), accessibility (ease of subscription to and utilization of the Text4Hope program), appropriateness (numbers of residents subscribing to the program), and effectiveness (6- and 12-week changes in the Perceived Stress, GAD-7, and PHQ-9 scales). It may also be possible to examine efficiency (cost avoidance and efficiencies through reduced need for face-to-face counselling) and safety (self-reports of decreased crisis and urgent service calls, and decreased emergency medical services utilization rates).

We will evaluate the efficacy of Text4Hope with the reductions of perceived stress, anxiety, and depression at 6 weeks and 12 weeks. Data analysis will include the standard use of parametric and nonparametric techniques (eg, within-subject general linear models), including multiple comparison Type 1 error corrections. Power analysis with effect sizes based on Agyapong group research publications [26-28,36,37] indicates a sufficient effect size for the expected Text4Hope program subscriber sample size. As the sample size for the data set generated from this project will be large, in addition to the conventional statistics used in these projects, we plan to adopt a “big data” analysis approach to examine data-driven patterns. Using machine learning and data mining methods, we expect to capture the most powerful window on differences and high-order sets of potential interactions. With our program targeting 300,000 Canadians to self-subscribe, with an expected 60,000 completing the survey, we are confident that this project has adequate power for our basic cross-sectional approach. We will develop predictive models using baseline measurements (features) and machine learning algorithms (eg, the least absolute shrinkage and selection operator [LASSO], support vector regression [SVR], and random forest regression, etc [38-40]) to predict the efficacy of our intervention at 6 and 12 weeks, as measured by the change in assessment scores (eg, GAD-7, PHQ-9) compared to the baseline. We will perform a 10-fold cross-validation to evaluate the performance of the models. During cross-validation, the data will be randomly segmented into 10 folds, with each fold containing 10% of the data. For each iteration, 1 fold will be left out as the testing data and the remaining 9 will be the training data. Within each training session, another internal 10-fold cross-validation will be used to select the best features, algorithm, and corresponding hyperparameters as the model to be trained on this specific training session. The selected model in the training data will then be applied to the testing data. This procedure is crucial for predictive tools as the data being tested has never been seen in any way by the model. The performance of the models will be evaluated using the Pearson correlation between the predicted and actual reduction of the outcome measures.

Due to the ongoing shifts in infection rates and public health measures over time, together with a shifting and unpredictable pattern of economic impacts, it will be important to include data on infection and death rates as well as the overall economic measures (including unemployment rates) associated with the baseline, 6-week, and 12-week measures. Such variables will be included in the machine learning analysis of the project data [41]. Including these variables will enable the model to account for variables that may influence expected rates of anxiety- and depression-related symptoms.

## Discussion

The impact of the COVID-19 global pandemic on health, way of life, and psychological safety and wellbeing is difficult to overstate. The psychological impact on the general population, both during and after the crisis, requires the use of innovative techniques that can serve the high number of people requiring support, while respecting the need to maintain physical distancing.

The current protocol describes the use of mobile health technology as a convenient, cost-effective, and accessible means for implementing a population-level psychological intervention during the pandemic. This program is empirically supported by previous research results, showing good outcomes as well as high user satisfaction [26,27]. This project will evaluate outcomes with standardized, empirically validated questionnaires, and will also provide key information regarding prevalence rates of stress, anxiety, and depression in the Canadian population during the COVID-19 pandemic; demographic correlates of this distress; and outcome data related to a scalable population-level intervention. Information from this study will thus be critical for practitioners, as well as useful for informing policy and decision-making regarding psychological interventions during the COVID-19 pandemic. If Text4Hope is effective for the Canadian population, we will explore scale-up and national implementation, and will disseminate this program for adaptation for potential global use through the APEC Digital Hub for Mental Health [42].

Limitations of this protocol include a lack of baseline data on stress, anxiety, and depression levels before self-isolation measures were implemented in Alberta; this was unavoidable as our study was initiated shortly after quarantine and self-isolation measures were introduced. Nonresponse bias may also affect the expected results, as program subscribers are a sample of the population, not the entire population of the province. Nonrespondents may differ in a systematic way compared to respondents. For example, they may differ in their baseline level of mental wellness, be more (or less) affected by the pandemic, or have limitations in literacy or English fluency. In view of the limitations noted above, any prevalence estimates must be interpreted with caution, and compared to the conventional baseline of subsequent conventional prevalence estimates. The authors also note that this protocol does not include a control group and this raises the question of specificity concerning hypothesis 3, which concerns reductions in perceived stress, anxiety, and depressive symptoms. Previous RCT work from this group has demonstrated the efficacy of supportive SMS text messaging in intervention groups, compared to control groups that did not receive supportive text messages; instead, the control group received the same survey requests as the intervention group in addition to a single text message every 2 weeks, thanking them for participating in the study [26,37]. Given the intention to provide support to the catchment population of this study and the prior evidence for efficacy, it would be unethical to include a control group in the current protocol. Nevertheless, prior studies did include subscriber satisfaction surveys, as does this protocol, and that measure will provide evidence for engagement of the subscribers with the program (simply put, if subscribers ignored the text messages, it is highly unlikely that there would be positive satisfaction survey results). In the current health implementation context of this protocol, the comparison of changes in outcome measures in relation to comparison with effect sizes from our prior work (together with an assessment of the degree of correspondence of subscriber satisfaction survey responses to changes in our outcome measures) will be a good indicator of subscriber engagement in the absence of a control condition. Despite these limitations and possible bias factors, our protocol will provide useful data about the mental health characteristics of individuals in the early stages of the COVID-19 pandemic. We expect that our results will represent an important initial source of information for government and health care planners in determining the nature and quality of services required to address mental health challenges arising during this pandemic, as well as future pandemics that employ self-isolation or quarantine measures. Specifically, planning for and implementing virtual care programs, including supportive SMS text messages, may be a fruitful approach to supporting isolated or quarantined individuals. In addition, we expect the supportive SMS text messaging intervention to have a positive effect on mental well-being and we will be able to measure this well within the expected sample size.

## Abbreviations

COVID-19: coronavirus disease
GAD-7: Generalized Anxiety Disorder-7 scale
LASSO: least absolute shrinkage and selection operator
PHQ-9: Patient Health Questionnaire-9
RCT: randomized controlled trial
SARS-CoV-2: severe acute respiratory syndrome coronavirus 2
SVR: support vector regression
